# Decolonization to reduce *Staphylococcus aureus* surgical site infections after hip or knee arthroplasty

**DOI:** 10.1017/ash.2022.315

**Published:** 2022-11-04

**Authors:** Natalia Everstz, Caroline Marshall, Matthew Richards, Steven Y.C. Tong

**Affiliations:** 1 Melbourne Medical School, Faculty of Medicine, Dentistry and Health Sciences, University of Melbourne, Melbourne, Victoria, Australia; 2 Infection Prevention and Surveillance Service, The Royal Melbourne Hospital, Melbourne, Victoria, Australia; 3 Victorian Infectious Diseases Service, The Royal Melbourne Hospital, at the Peter Doherty Institute for Infection and Immunity, Melbourne, Victoria, Australia; 4 Department of Infectious Diseases, The University of Melbourne at the Peter Doherty Institute for Infection and Immunity, Melbourne, Victoria, Australia

Surgical site infection (SSI) following joint replacement surgery, may lead to implant failure, higher morbidity and mortality, prolonged hospitalization and increased costs.^1^ *Staphylococcus aureus* is the leading cause; it is responsible for half of all SSIs following hip or knee arthroplasty.^2^ Infection with methicillin-resistant *S. aureus* (MRSA) is associated with greater morbidity and mortality than methicillin-susceptible *S. aureus* (MSSA).^3^

*S. aureus* is a normal skin and mucosal flora and asymptomatically colonizes up to one-third of the population.^4^ SSI risk is significantly increased if patients are colonized with *S. aureus.* ^5^ Reducing colonization prior to surgery is considered a key measure to reducing SSI incidence. *S. aureus* screening and decolonization programs are effective in reducing SSI incidence and are widely used.^6^

We investigated the contribution of a screening and decolonization protocol prior to hip or knee arthroplasty in reducing the incidence of *S. aureus* joint infections.

## Methods

We identified patients undergoing hip or knee arthroplasty at the Royal Melbourne Hospital, Melbourne, Australia, between January 1, 2012, and July 8, 2016, by extracting data from surveillance data collected by the infection prevention service. SSIs were defined as any wound-site infection involving skin, subcutaneous tissue, and/or deep soft tissue within 90 days of the procedure.^7^

Patients scheduled for elective prosthetic hip or knee arthroplasty attended the preadmission clinic for screening groin and nasal swabs. If *S. aureus* was not detected on screening swabs, no decolonization treatment was prescribed. Patients positive for *S. aureus* were instructed to apply 2% mupirocin ointment to both nostrils twice daily and to shower each day using 2% chlorhexidine skin cleanser for the 5 days immediately prior to surgery. Emergency patients were not screened or decolonized unless screening had occurred in anticipation of elective surgery.

Patients were given antibiotic prophylaxis prior to surgery with cefazolin (1 g for <80 kg and 2 g for ≥80 kg, commencing within 1 hour of incision, with a repeat dose for surgery >4 hours) or vancomycin (1.5 g intravenously, commencing 30–120 minutes prior to incision).

## Results

### Cohort

Between January 1, 2012, and July 8, 2016, 1,268 patients underwent hip or knee prosthetic joint surgery (Supplementary Fig. 1). The mean age of this cohort was 71.4 years, and 63% were female.

### Colonization

*S. aureus* colonization was detected in 256 (34%) of 754 screened patients; 242 (32%) were colonized with MSSA and 14 (2%) were colonized with MRSA. Patients undergoing revision surgery (n=128) had a MRSA colonization rate of 3% (n = 4) compared to 1% (n = 10) for those undergoing primary surgery (*P* = .45).

### Surgical-site infections

**Fig. 1.**
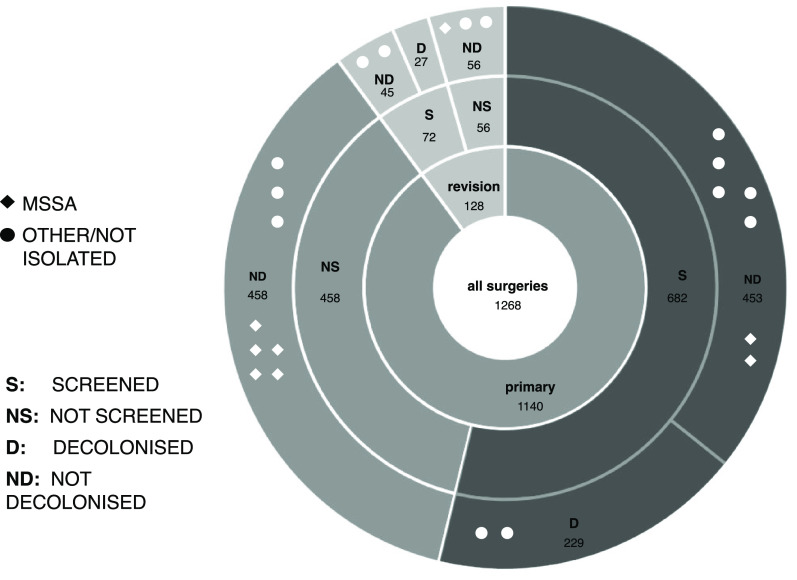
Incidence of surgical-site infection (SSI) among different cohorts of surgical patients. The symbols represent organisms causing infection: methicillin-sensitive *Staphylococcus aureus* (MSSA); organisms other than *S. aureus* or organisms that could not be cultured (other or not isolated). Between the January 1, 2012, and July 8, 2016, 1,268 patients underwent hip or knee prosthetic joint surgery. Of those, 754 were screened and 256 were decolonized. Of 453 patients screened but not decolonized, 7 primary surgical patients went on to develop an SSI (1.5%). The revision surgery group had the highest incidence for infection overall, with 5 patients (3.9%) who developed infection among 128 patients. The decolonized group had the lowest incidence of infection; no infection with *S. aureus* was observed in the decolonized group. No difference was observed in the frequency of infection due to other species between the decolonized and non-decolonized groups (0.8%; cf, 1.0%).

Of 1,268 patients, 22 (2%) developed infections, of whom 8 (36%) had *S. aureus* (all MSSA) (Supplementary Table 1). Elective revision surgeries had the highest infection rate of any cohort (4.0%, n = 4 of 99), and elective primary surgeries had the lowest infection rate (1.3%, n = 12 of 906; *P* = .06) (Supplementary Table 1). No patients colonized with *S. aureus* at screening (and hence prescribed the decolonization protocol) developed infections due to *S. aureus* (0%), compared to 8 *S. aureus* infections in the non-decolonized group (0.8%; *P* = .37) (Fig. 1).

Because non-decolonized patients were more likely to have had emergency surgery and a different risk profile (Supplementary Table 2), we also restricted analysis to the elective primary surgery subgroup in which the decolonized and non-decolonized patient groups had similar risk factors for SSI (Supplementary Table 3). In this group of 906 patients, 10 (1.5%) of 679 non-decolonized patients developed infection (4 with *S. aureus*, 4 with other organisms, and 2 with no organism isolated) compared to 2 (0.9%) of 227 decolonized patients (no *S. aureus* but 2 other organisms) (*P* = .74 for any infection and *P* = .58 for *S. aureus* infections only) (Supplementary Table 4 online). Notably, of those who were screened and in whom *S. aureus* had not been detected (n = 446), there were 2 *S. aureus* infections.

## Discussion

We observed no *S. aureus* infections among decolonized patients even though they were all previously colonized with *S. aureus*, a major independent risk factor for *S. aureus* infections in patients undergoing joint replacement surgery.^6^

Studies already support the role of screening followed by decolonization of *S. aureus*–colonized patients.^6,8^ Our results also suggest that a universal *S. aureus* decolonization program without initial screening should be considered. There are several issues with screening first and decolonizing only those who are *S. aureus* colonized. First, many patients do not receive screening. In our setting, only 59% of patients underwent screening prior to their arthroplasty procedure. Second, screening is not 100% sensitive for *S. aureus* carriage and depends on the anatomical sampling site and the timing of sampling.^9^ Patients not colonized at the time of screening may be colonized at the time of surgery. Of 498 patients who were screened and in whom *S. aureus* was not detected, there were 2 subsequent *S. aureus* infections. Third, screening is resource intensive. Compared to preoperative screening and decolonization, universal decolonization has been shown to be more cost-effective.^10^

This study had several limitations. *S. aureus*–colonized patients were prescribed the decolonization protocol, but we did not collect data on protocol adherence or whether carriage was cleared. However, poor adherence or failure of decolonization would have undermined the effectiveness of the intervention; nevertheless, no *S. aureus* infections occurred in the decolonized cohort. Our data were from only a single institution, numbers of infections were low, and the study was observational. These results may not be generalizable to other institutions, and we were not able to demonstrate a statistically significant reduction in infection rates.

With this decolonization protocol, no *S. aureus* infections occurred after arthroplasty, even among previously colonised patients.

## Supplementary material

For supplementary material accompanying this paper visit https://doi.org/10.1017/ash.2022.315. click here to view supplementary material
