# Supplementary material for: Decolonization to reduce Staphylococcus aureus surgical site infections after hip or knee arthroplasty
Source: Antimicrob Steward Healthc Epidemiol. 2022 Nov 4;2(1):e179. doi: 10.1017/ash.2022.315 (PMC9641506; doi:10.1017/ash.2022.315)
Supplement: Supplementary file 1 [file S2732494X22003151sup001.docx]

**Supplementary Methods**

We included elective and emergency primary and revision procedures. Primary procedures are where a surgical prosthesis is being implanted for the first time, and revision procedures are a revision of a previously implanted prosthesis, including where an existing prosthesis was removed and replaced. We excluded revision procedures due to infection. We considered screening results obtained up to six months prior to surgery valid for that surgery. Superficial-incisional, deep-wound and organ/space infections were defined according to VICNISS criteria (based on Centers for Disease Control (CDC) National Healthcare Safety Network (NHSN) criteria).^18^

Nasal swabs were taken using Copan ESwabs containing liquid Amies media. One swab was used to sample both nostrils and another was used to swab both sides of the groin and each was separately plated onto chromogenic media (Brilliance MRSA, Oxoid) and Horse Blood Agar (Thermofisher). Typical colonies were selected and identification confirmed with matrix-assisted laser desorption/ionization- time of flight (MALDI-TOF) (Bruker). Susceptibility testing was performed using Vitek 2 (Biomerieux, Marcy L’Etoile, France) according to CLSI interpretive breakpoints and criteria. Final results were available in 24-48 hours.

Antibiotic prophylaxis prior to surgery was recommended for patients with current or a history of MRSA colonisation or infection, who were in hospital for ≥5 days before surgery, undergoing a revision procedure or who were beta-lactam allergic, according to hospital procedure.

Data are presented descriptively using summary statistics including means, standard deviations and interquartile ranges. SSI rates in decolonised and non-decolonised patients were compared for the entire cohort, and for that of primary elective surgeries. Within both, the decolonised and non-decolonised groups were compared by age, sex and ASA score. Data were analysed using Microsoft Excel version 15.32 and Stata 15.1 (College Station, Texas). Differences in proportions were compared using chi square or Fisher’s exact tests.

**Supplementary Figures**

**Supplementary figure 1**: Surgical patient cohort and subcohorts. Surgeries were divided among elective and emergency surgeries. These were then further subdivided into primary and revision surgeries. Primary surgeries are those where a surgical prosthesis is being implanted for the first time. Revision surgeries include revisions of previously implanted prostheses, and removals of existing prostheses accompanied by a replacement. The numbers accompanying the figure indicate the number of patients within each respective cohort, and the percentage of total patients (n=1268) within each cohort. Due to rounding, not all percentages add to 100%.

**Supplementary Tables**

**Supplementary Table 1**: Infections in elective and emergency surgery cohorts

|  | ***S. aureus* infection**  **(n,%)** | **Infection due to other/**  **unidentified organism**  **(n,%)** | **Total infection**  **(n,%)** |
| --- | --- | --- | --- |
| Elective (n=1005) | 5 (0.5%) | 11 (1.1%) | 16 (1.6%) |
| Primary (n=906) | 4 (0.4%) | 8 (0.9%) | 12 (1.3%) |
| Revision (n=99) | 1 (1.0%) | 3 (3.0%) | 4 (4.0%) |
| Emergency (n=263) | 3 (1.1%) | 3 (1.1%) | 6 (2.3%) |
| Primary (n=234) | 3 (1.3%) | 2 (0.9%) | 5 (2.1%) |
| Revision (n=29) | 0 (0.0%) | 1 (3.4%) | 1 (3.4%) |
| Total (n=1268) | 8 (0.6%) | 14 (1.1%) | 22 (1.7%) |

**Supplementary table 2**: Demographic descriptors of the non-decolonised and decolonised groups across all surgeries

|  | **NOT DECOLONISED**  **N=1012** | **DECOLONISED**  **N=256** |
| --- | --- | --- |
| SURGERY TYPE (n, %) |  |  |
| Elective primary surgery | 679 (67) | 227 (89) |
| Revision surgery (primary and emergency) | 101 (10) | 27 (11) |
| Emergency Surgery | 260 (26) | 3 (1) |
| DEMOGRAPHICS |  |  |
| Mean age (years, SD) | 72.6 (12.7) | 66.4 (12.8) |
| Sex (Female, %) | 678 (67) | 144 (56) |
| ASA SCORE (n, %) |  |  |
| ASA < 4 | 891 (88) | 244 (95) |
| ASA ≥ 4 | 95 (9) | 5 (2) |
| Not specified | 26 (7) | 7 (3) |

ASA 1: Patient completely healthy; ASA 2: Patient with mild systemic disease; ASA3: Patient with severe systemic disease; ASA 4: Patient with incapacitating disease that is a constant threat to life; ASA 5: Moribund patient who is not expected to survive

**Supplementary table 3**: Demographic descriptors of the non-decolonised and decolonised groups in the elective primary surgery cohort

|  | **Not Decolonised**  **N=679** | **Decolonised**  **N=227** |
| --- | --- | --- |
| DEMOGRAPHICS |  |  |
| Mean age (years, SD) | 70.2  (12.2) | 66.1  (12.7) |
| Sex (Female, %) | 469 (69) | 127 (56) |
| ASA Score (n, %) |  |  |
| ASA < 4 | 631 (93) | 218 (96) |
| ASA ≥ 4 | 29 (4) | 2 (1) |
| Not specified | 19 (3) | 7 (3) |

**Supplementary Table 4**: Infections in decolonised and non-decolonised groups, among elective primary and elective revision surgery cohorts

|  | **Primary**  **n=906** | | **Revision**  **n=99** | | **Combined**  **n=1005** | |
| --- | --- | --- | --- | --- | --- | --- |
|  | Not decolonised | Decolonised | Not decolonised | Decolonised | Not decolonised | Decolonised |
| *S. aureus infection* | 4  (0.6%) | 0  (0%) | 1  (1.4%) | 0  (0%) | 5  (0.7%) | 0  (0%) |
| Other infection | 6  (0.7%) | 2  (0.9%) | 3  (3.0%) | 0  (0%) | 9  (1.2%) | 2  (0.8%) |
| No infection | 669  (98.5%) | 225  (99.1%) | 69  (94.5%) | 26  (100%) | 738  (98.1%) | 251  (99.2%) |
